# Anticancer drugs repurposed for Alzheimer’s disease: a systematic review

**DOI:** 10.1186/s13195-021-00831-6

**Published:** 2021-05-05

**Authors:** Antonio Ancidoni, Ilaria Bacigalupo, Giulia Remoli, Eleonora Lacorte, Paola Piscopo, Giulia Sarti, Massimo Corbo, Nicola Vanacore, Marco Canevelli

**Affiliations:** 1 National Center for Disease Prevention and Health Promotion, Italian National Institute of Health, Via Giano della Bella 34, 00162 Rome, Italy; 2 Department of Neuroscience, Italian National Institute of Health, Viale Regina Elena, 299, 00161 Rome, Italy; 3 Department of Human Neuroscience, Sapienza University, Rome, Italy; 4 Department of Neurorehabilitation Sciences, Casa Cura Policlinico, Via Dezza 48, 20144 Milan, Italy

**Keywords:** Alzheimer’s disease, Cancer, Anticancer drugs, Clinical trials, Drug repositioning

## Abstract

**Background:**

The relationship between cancer and dementia is triggering growing research interest. Several preclinical studies have provided the biological rationale for the repurposing of specific anticancer agents in Alzheimer’s disease (AD), and a growing number of research protocols are testing their efficacy and safety/tolerability in patients with AD.

**Methods:**

The aim of the present systematic review was to provide an overview on the repurposing of approved anticancer drugs in clinical trials for AD by considering both ongoing and completed research protocols in all phases. In parallel, a systematic literature review was conducted on PubMed, ISI Web, and the Cochrane Library to identify published clinical studies on repurposed anticancer agents in AD.

**Results:**

Based on a structured search on the ClinicalTrials.gov and the EudraCT databases, we identified 13 clinical trials testing 11 different approved anticancer agents (five tyrosine kinase inhibitors, two retinoid X receptor agonists, two immunomodulatory agents, one histone deacetylase inhibitor, and one monoclonal antibody) in the AD continuum. The systematic literature search led to the identification of five published studies (one phase I, three phase II, and one phase IIb/III) reporting the effects of antitumoral treatments in patients with mild cognitive impairment or AD dementia. The clinical findings and the methodological characteristics of these studies are described and discussed.

**Conclusion:**

Anticancer agents are triggering growing interest in the context of repurposed therapies in AD. Several clinical trials are underway, and data are expected to be available in the near future. To date, data emerging from published clinical studies are controversial. The promising results emerging from preclinical studies and identified research protocols should be confirmed and extended by larger, adequately designed, and high-quality clinical trials.

## Background

Cancer and dementia, including Alzheimer’s disease (AD), represent two of the leading causes of mortality and disability worldwide [1]. Although these pathological conditions have traditionally been associated with distinct pathophysiological mechanisms and phenotypic manifestations, a growing body of research has recently been focused on their possible mutual relationship [2, 3].

Some studies suggested an inverse relationship between cancer and dementia (mostly of the AD type), with cancer history decreasing the risk of AD and patients with AD having a lower probability of developing cancer [4]. However, it is crucial to clarify the either genetic or molecular mechanisms that could be somehow at crossroads between these two conditions and sustain their possible negative association. In contrast, other studies provided preliminary evidence that cancer and AD may share some common pathways. In this regard, a recent study analyzed all biological hallmarks of cancer in the AD literature and concluded that not all cancer etiopathogenetic events run the opposite direction in AD [5]. Moreover, since Hanahan and Weinberg updated their research on the hallmarks of cancer [6, 7], there is accumulating evidence that these key molecular pathways may also affect the risk, onset, and progression of AD and that some specific hallmarks can actually be common to these diseases [8].

For instance, it has been shown that some oncoproteins, such as protein kinases, are dysregulated in AD, since hyperphosphorylation of neurofibrillary tangles is one of the distinctive features of AD [8]. Another cancer hallmark, namely inflammation [6, 7, 9], is also increasingly invoked to explain the neuropathological changes leading to AD. Indeed, the activation of microglia and astrocytes and the resulting neuroinflammation are currently considered as major events in the pathophysiology of this neurodegenerative condition [10, 11] and it is demonstrated that amyloid plaques are surrounded by activated microglia both in early and late stages of the disease [12]. Targeting these immune responses could therefore represent an alternative therapeutic strategy in AD [13, 14]. Finally, other biological processes and abnormalities, such as genome instability and deregulation of cellular energetics, probably constitute common underlying mechanisms [5].

The therapeutic implications of the complex relationship between cancer and dementia have instead been poorly investigated yet. Given the current therapeutic gap in AD, the scientific community is growingly investigating whether drugs approved for other diseases may be repurposed to slow down or even hamper AD course [15, 16]. In this regard, some anticancer drugs have been shown to have a good permeability through the blood-brain barrier (BBB), thus potentially exerting relevant effects against AD pathology [17, 18]. A recent retrospective study of approximately 3.5 million older American veterans showed that cancer treatment was independently associated with decreased AD risk and that those who received chemotherapy had a lower risk than those who did not [19]. Accordingly, in a study of nearly 62,000 older women diagnosed with breast cancer, the risk of developing AD and other dementias was significantly lower in patients receiving chemotherapy [20]. In addition, some studies suggest that anticancer drugs may also act as disease-modifying therapies once the AD-related neurodegenerative process has already started [21]. Based on these preliminary findings, a growing number of research protocols are testing the efficacy and safety of approved anticancer agents in patients with AD.

Hence, the aim of the present systematic review was to provide an overview on the repurposing of approved anticancer drugs in clinical trials for AD. Both ongoing research protocols and published studies were considered for this purpose. Furthermore, attention was paid to methodological and reporting quality.

## Materials and methods

### Systematic review of ongoing research protocols

Two databases were used as sources for the present study: (i) the ClinicalTrials.gov for studies registered in the USA and (ii) the EudraCT (European Union Drug Regulating Authorities Clinical Trials Database) for all interventional studies registered in the European Union. The two databases were investigated in December 2020, to identify ongoing research protocols testing anticancer agents in the AD continuum by using both the following search terms: “Alzheimer OR Dementia.” No restriction was applied for recruitment phase/status, study design, and study phase. Two reviewers (AA, EL) independently selected protocols deemed to be eligible for the review topic. Specifically, only studies (i) investigating pharmacological compounds approved by national or international drug agencies (e.g., Food and Drugs Administration, European Medicines Agency) as anticancer agents and (ii) recruiting patients with a clinical diagnosis of AD or mild cognitive impairment (MCI) or assessing AD biomarkers in subjects with preclinical AD and healthy volunteers were selected. Trials focusing on neurodegenerative dementias other than AD (i.e., Lewy body dementia, Parkinson’s disease dementia, frontotemporal dementias) were instead not considered for the present analysis. Any disagreement in the protocols’ selection was resolved by discussion between the authors. For each selected trial, the main methodological and clinical information (IDs, status, duration, intervention, sample size, sociodemographic and clinical characteristics of participants, relevant inclusion and exclusion criteria, diagnosis at the baseline, primary and secondary endpoints) were extracted in standardized forms. Along with this information, it was investigated whether the tested drugs were used as disease-modifying or as symptomatic treatments.

### Literature search of published clinical studies

The literature review was performed following the methodology described in the Cochrane handbook for systematic reviews [22] and was reported based on the PRISMA statement for reporting systematic reviews and meta-analyses [23]. A systematic literature search was conducted in the biomedical databases, i.e., PubMed, ISI Web of Knowledge, and the Cochrane Library to identify published clinical trials testing approved anticancer agents in AD. The search was updated to January 2021. The following terms were used: (Alzheimer* OR dementia*) AND (((cancer* OR neoplas* OR tumor* OR oncolog* OR anticancer* OR anti-cancer* OR anti-neoplas* OR antineoplas* OR tumor* OR antitumor* OR anti-tumor*) AND (drug* OR treatment* OR therap*) AND (“clinical trial” OR “clinical trials” OR “randomized trial” OR “randomised trial” OR “randomized trials” OR “randomised trials”)) OR (bexaroten* OR Nilotinib OR AMN107 OR Dasatinib OR Daratumumab OR Tamibarotene OR OAM80 OR Thalidomide OR Lenalidomide OR Masitinib OR AB1010 OR Bosutinib OR PF-5208763 OR Ski-606 OR Pexidartinib OR PLX3397 OR Vorinostat)). Specific drug names and/or codes included in the search string were selected based on the trials identified by the search in the ClinicalTrial.gov and EudraCT databases.

**Fig. 1:**
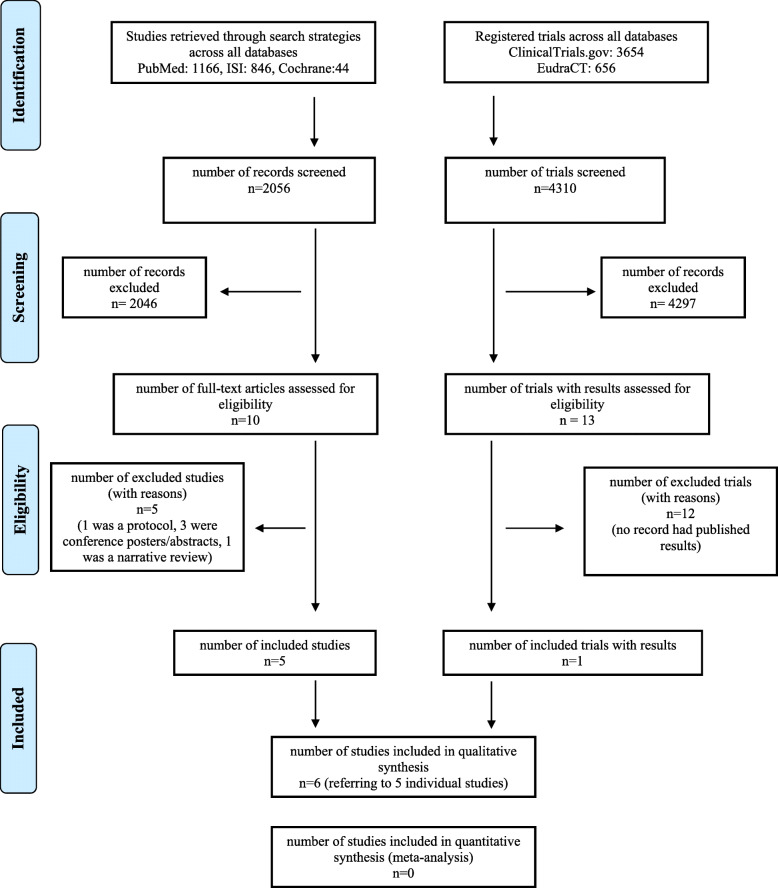
Modified PRISMA flow diagram for clinical trial selection

Studies were independently selected by four reviewers (AA, EL, IB, GR) based on their pertinence with and relevance to the topic of the review. Disagreements were resolved by consensus. Only clinical studies (i) investigating approved anticancer agents and (ii) enrolling patients with a clinical diagnosis of AD (of any severity) or MCI or exploring the effect of anticancer agents on AD biomarkers in participants with preclinical AD or healthy subjects were selected. Preclinical studies, study protocols, and reviews as well as studies recruiting participants without a diagnosis of AD were not considered. Studies that published only trial protocols and/or patients’ baseline features were excluded. Conference proceedings, abstracts, posters, letters, and editorials were also excluded. When trial results were available both from clinical trials databases and publications, data were compared to identify possible discrepancies. A modified PRISMA Flow Diagram was used to report the flow process for study selection (Fig. 1). Then, the Cochrane risk-of-bias tool for randomized trials (RoB) was applied to published trial studies for methodological and quality assessment. The RoB tool is suitable for individually randomized, parallel-group, and cluster-randomized trials. The qualitative assessment of included studies was performed using the software Review Manager, version 5.3, developed by the Cochrane Collaboration.

## Results

### Overview of identified research protocols

A total of 3654 protocols registered on ClinicalTrials.gov and 656 protocols registered on EudraCT were identified and screened. Among them, 13 studies fulfilled the selection criteria as they were testing approved anticancer agents in samples of patients in the AD continuum (Fig. 1). Eleven of these studies were only registered in ClinicalTrials.gov. One trial was registered on both databases, and one protocol was registered exclusively in the EU database. Three phase I, one phase I/II, eight phase II, and one phase IIb/III protocols were identified (Table 1).

**Table 1:** IDs, intervention, main features, and outcomes of selected trial protocols

Identifier	Intervention	Duration	Placebo	Estimated enrollment	Age	Diagnosis	MMSE at baseline	Primary outcome	Secondary outcome	Status
Phase I										
NCT03056495	Vorinostat	4 weeks	N	44	≥ 55 to ≤ 90	Mild AD	≥ 22 to ≤ 27	Maximum-tolerated dose	Incidence of treatment emergent AEs Pharmacokinetics Pharmacodynamics	Recruiting
NCT02921477	Bosutinib	1 year	N	64	≥ 45 to ≤ 89	MCI to moderate dementia	–	Safety, tolerability	–	Enrolling by invitation
NCT02061878	Bexarotene	5 days	Y	12	≥ 21 to ≤ 50	Healthy volunteers with the ApoE ε3/ε3 genotype	–	CSF levels of ApoE and Aβ clearance	Fractional clearance rate of beta-amyloid peptide in CNS	Completed
Phase I–II										
NCT04063124	Dasatinib (+quercetin)	12 weeks	N	5	≥ 65	Clinical diagnosis of AD	–	Brain penetrance after 12 weeks	CSF-tau, CSF-amyloid beta, CSF-IL-6, CSF-P16, MoCA Electronic gait mapping under single- and dual-task conditions	Recruiting
Phase II										
NCT04070378	Daratumumab	16 weeks	N	15	≥ 55 to ≤ 85	Mild to moderate AD	≥ 15 to ≤ 26	ADAS-Cog/11	ADAS-Cog/12, MMSE, ADAS-ADL CDR-SOB, ADCOMS	Recruiting
NCT02947893	Nilotinib	1 year	Y	42	≥ 50	Mild to moderate AD	≥ 17 to ≤ 24	Safety, tolerability Pharmacokinetics	Abl inhibition to demonstrate CNS target engagement	Active, not recruiting
NCT04032626	Lenalidomide	12 months of treatment followed by 6 months of washout. The trial will last 20 months in duration.	Y	30	≥ 50 to ≤ 89	MCI	≥ 22 to ≤ 28	ADAS-Cog ADCS-ADL CDR-SOB MMSE	AEs and SAEs Change in brain amyloid loads Change in blood inflammatory markers Change in neurodegeneration	Recruiting
NCT01120002	Tamibarotene	–	Y	50	≥ 55 to ≤ 80	Mild to moderate AD	≥ 10 to ≤ 26	Changes in ADAS-JCog (Japanese version)	MMSE, ADCS-ADL CIBIC-Plus	Unknown
NCT01782742	Bexarotene	4 weeks	Y	20	≥ 50 to ≤ 90	Probable AD	≥ 10 to ≤ 20	Change in the composite amyloid burden of the brain according to ApoE genotype	MMSE, ADAS-Cog NPI, CDR ADCS-ADL Serum level change of Aβ42 and Aβ40 (all subjects and ApoE-ε4 noncarriers) Change in Aβ42/Aβ40 ratio (all subjects and ApoE-ε4 noncarriers)	Completed
2016-000429-38	Pexidartinib	16 weeks	Y	–	≥ 55 to ≤ 85	Mild to moderate AD	≥ 16 to ≤ 26	Safety and tolerability	Effect on microglia inflammation Cognitive and functional measures (tests not reported) Pharmacokinetics (CSF to plasma ratio) CSFR-1 biomarkers in blood	Prematurely ended
NCT00976118	Masitinib	24 weeks	Y	34	≥ 50	Mild to moderate AD	≥ 12 to ≤ 26	ADAS-Cog	CIBIC-Plus CDR MMSE	Completed
NCT01094340	Thalidomide	24 weeks	Y	20	≥ 50 to ≤ 90	Probable AD	≥ 12 to ≤ 26	Improve cognition	Improve cognition	Unknown
Phase II/III										
NCT01872598 (2010-021218-50)	Masitinib	Minimum of 6 months	Y	721	≥ 50	Diagnosis of AD	≥ 12 to ≤ 25	ADAS-Cog ADCS-ADL	MMSE, CIBIC-Plus	Completed

**Fig. 2:**
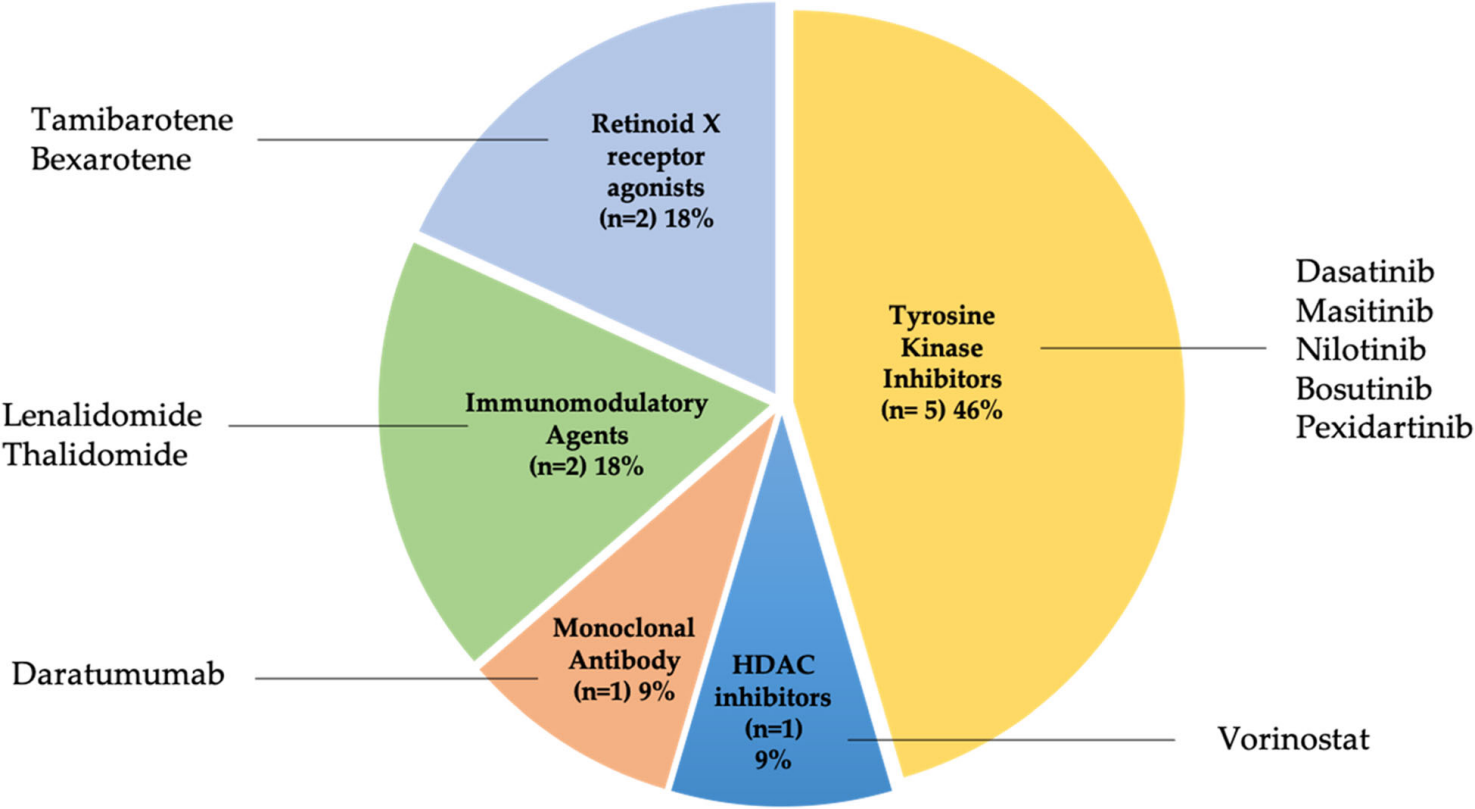
Pie chart of approved anticancer drugs in trials for Alzheimer’s disease

Overall, 11 different approved anticancer drugs were investigated. Five drugs (bosutinib, dasatinib, masitinib, nilotinib, and pexidartinib) belong to the class of tyrosine kinase inhibitor (TKI) class, two are immunomodulatory agents (lenalidomide and thalidomide), two are retinoid X receptor (RXR) agonists (bexarotene and tamibarotene), one is a monoclonal antibody (daratumumab), and one is a histone deacetylase (HDAC) inhibitor (vorinostat) (Fig. 2). A comprehensive overview of the role of these drugs in cancer, their regulatory approved indications, and the rationale for their therapeutic potential for AD is provided in Table 2 [24–47, 49, 50].

**Table 2:** Anticancer drug class, mechanism of action, approved indications, and therapeutic rationale for repurposing in Alzheimer’s disease

Molecule	Drug class	Role in cancer	Approved indication(s)	Rationale for therapeutic purpose in AD	Reference
Vorinostat	HDAC inhibitor	Antiproliferative effect through modulation of histone acetylase activity	Cutaneous manifestations in cutaneous T cell lymphoma	Restoration of synaptic plasticity. Improved memory long-term potentiation, reduction in Aβ and tau pathology.	[24, 25]
Bosutinib	Tyrosine kinase inhibitor	The primary target is the BCR-ABL kinase. Inhibition of several tyrosine kinases	Ph+ chromosome chronic myeloid leukemia	Increase in blood and brain IL-10 and soluble CX3CL1	[26–29]
Masitinib	Tyrosine kinase inhibitor	Inhibition of the receptor tyrosine kinase c-Kit. Inhibition of PDGFR, Lck, FAK, and FGFR3	Mast cell tumor (for veterinary use)	Inhibition of c-Kit receptor in MCs. It is capable of blocking Fyn that is involved in tau phosphorylation. Cognitive improvements as a result of Fyn inhibition	[30–34]
Dasatinib	Tyrosine kinase inhibitor	Inhibition of BCR-ABL, SRC family kinases, c-Kit, EPHA2, and PDGFRβ	Ph+ chromosome chronic myeloid leukemia in chronic phase and acute lymphoblastic leukemia in blastic phase	Removal of senescent cells from the plaque environment. Inhibition of amyloid-dependent microgliosis	[35, 36]
Nilotinib	Tyrosine kinase inhibitor	Antiproliferative effects through inhibition of several kinases (BCR-ABL, c-Kit and PDGF, PI3K-Akt, JACK-STAT)	Ph+ chromosome chronic myeloid leukemia	Abl inhibition facilitates amyloid clearance and reduces inflammation. Upregulation of soluble CX3CL1	[26–28]
Pexidartinib	Tyrosine kinase inhibitor	It works by inhibiting the colony-stimulating factor (CSF1)/CSF1 receptor pathway.	Symptomatic tenosynovial giant cell tumor	Reduction in microglial neuroinflammation	[37–40]
Bexarotene	Retinoid X receptor agonist	Inhibition of cell cycle progression, prevention of multidrug resistance, inhibition of angiogenesis and metastasis	Advanced cutaneous T-cell lymphoma	Alter the CSF levels of ApoE Inhibition of Aβ42 aggregation	[21, 41–44]
Tamibarotene	Retinoid X receptor agonist	Specific agonist for retinoic acid receptor alpha/beta with possible binding to retinoid X receptors (RXR)	Relapsed or refractory acute promyelocytic leukemia (only in Japan)	Decreased insoluble Aβ 42 deposition in and increased VAChT and ACh in the brain and reduction of neuroinflammation	[45]
Thalidomide	Immunomodulatory agent	Possible anti-TNF-α effects. It may act as a VEGF inhibitor.	Multiple myeloma	Reduction of Αβ, inhibition of the expression of BACE1 enzyme. Reduction of proinflammatory TNF-α	[46]
Lenalidomide	Immunomodulatory agent	Tumor cell apoptosis by inhibition of bone marrow stromal cell support, by anti-angiogenic, anti-osteoclastogenic effects, and by immunomodulatory activity	Multiple myeloma; mantle cell lymphoma; follicular lymphoma	Reduction of the expression of TNF-α, IL-6, IL-8 Increase the expression of anti-inflammatory cytokines.	[47, 48]
Daratumumab	Monoclonal antibody	Targeting and induction of apoptosis in cells that highly express CD38	Relapse/refractory Multiple myeloma	AD pathology is attenuated in CD38-deficient mouse model	[49, 50]

Five protocols are currently active (nilotinib, lenalidomide, dasatinib, daratumumab, and vorinostat), four are completed (bexarotene and masitinib), one is enrolling by invitation (bosutinib), two are currently in unknown status (tamibarotene, thalidomide), and one is prematurely ended (pexidartinib). In terms of the number of trials identified, bexarotene and masitinib were the most represented agents that are being investigated in two trials each.

Concerning the study design, four studies (vorinostat, bosutinib, dasatinib, and daratumumab) are adopting a single-group assignment (i.e., no placebo) whereas nine are parallel-group, placebo-controlled studies.

Notably, only for one protocol, the study design and findings were already published in a journal [51]. No discrepancies between the registered protocol and the study publication were noticed regarding baseline characteristics, outcomes, and observed adverse events (AEs). A total of 1057 (range 5–721) subjects were planned to be enrolled in the considered protocols. The largest number of participants are expected to be recruited in the two trials with masitinib (*n*=756). Most studies focused on subjects with a diagnosis of MCI and mild to moderate AD (MMSE range 10–28). Only in one study, healthy volunteers were enrolled (bexarotene, NCT02061878). The duration of the planned interventions ranged between 5 days and 1 year.

Five trials (i.e., daratumumab, tamibarotene, lenalidomide, and both masitinib studies) adopted the Alzheimer’s Disease Assessment Score–Cognitive Subscale (ADAS-Cog) as the primary outcome.

Phase III masitinib and lenalidomide trials indicated the Alzheimer’s Disease Collaborative Study-Activities of Daily Living (ADCS-ADL) as the primary endpoint. The Mini Mental State Examination (MMSE) was indicated as the primary outcome in the lenalidomide study and as the secondary outcome in the daratumumab, tamibarotene, bexarotene, and masitinib studies. The Montreal Cognitive Assessment (MoCA) was used as the secondary endpoint in the dasatinib trial. In the phase I bexarotene study, where healthy volunteers were enrolled, only amyloid biomarkers were considered as both primary and secondary outcomes. No clinical outcomes were defined in phase I and phase I–II studies (vorinostat, bosutinib, bexarotene, and dasatinib).

### Overview of published clinical studies

The structured bibliographic searches yielded 2056 records. A total of 10 studies were selected based on their pertinence and relevance to the topic of the review. When applying the predefined inclusion and exclusion criteria, five studies were further excluded, with five studies to be included in the qualitative analysis [48, 51–54] (Fig. 1). Four phase II and one phase I studies were identified. For one study, results were also posted on ClinicalTrials.gov as mentioned in the previous section. Anticancer drugs for which a publication was available were bexarotene [52, 54], masitinib [51], nilotinib [53], and thalidomide [48]. The main characteristics and outcomes of the identified studies are summarized in Table 3.

**Table 3:** Main features of published clinical studies: study design, intervention, safety profile, and outcomes

Reference	Study design	Study population	Randomization	Objective(s)	Treatment duration	Intervention	AEs/SAEs	Number of dropouts	Achievement of endpoints
Cummings et al. [52]	Phase II, proof-of-concept randomized double-blinded, parallel-group, placebo-controlled single-site study	Treatment group: male 6/female 10 Age 74.9 ±6.6 Placebo group: male 1/female 3 Age 78.1 ±8.0 NINCDS-ADRDA criteria for AD Positive amyloid PET Average MMSE *B*, 13.7 *P*, 17.0	4:1 (*n*=20) 16 bexarotene 4 placebo	Drug-placebo change from baseline to week 4 of composite Aβ burden of the brain Change in cognitive scores from baseline to week 4 (MMSE, ADAS-Cog, ADCS-ADL, NPI, CDR-SOB) Change in Aβ40 and Aβ42 serum levels	4 weeks	Bexarotene (150mg/d) for 7d followed by 300mg/d from day 8 to 28	15/20 had increases in triglyceride levels (>200mg/dl) and cholesterol levels (>300mg/dl)	1 discontinued due to elevated triglyceride levels All controls completed the study	1. Significant reduction in composite amyloid burden in ApoE-ε4 noncarriers 2. No cognitive improvements
Piette et al. [51]	Phase II, multicenter, randomized double-blinded, placebo-controlled study	Treatment group: male 11/female 15 Age 72 ±12 Placebo group: male 2/female 6 Age 78 ±11 Mild to moderate AD (NINCD-ADRDA) Median MMSE score M, 19.1 P, 18	5:5:3 (*n*=34) 12 masitinib 3mg/kg/d 14 masitinib 6mg/kg/d 8 placebo	Improvement defined as a decrease ≥ 4 in ADAS-Cog Improvement defined as an increase in ADCS-ADL ≥ 3, CIBIC-Plus, CDR, and MMSE Safety	24 weeks	Masitinib (3 to 6mg/kg/d)	AEs (M), 65% (*n*=17) AEs (P), 38% (*n*=3) SAEs (M), 15% (*n*=4) SAEs (P), 13% (*n*=1)	21 prematurely ended: 9 adverse events (M) 2 protocol violation (1M; 1 P) 2 withdrawal of consent (M) 8 investigator death (7M; 1 P)	1. ADAS-Cog worsening at 12 and 24 weeks (6% in masitinib, 50% in placebo, *p*=0.04; *p*=0.046) 2. ADCS-ADL improvement at 12 weeks (50% in masitinib, 0% in placebo, *p*=0.05) Improvements not statistically significant at 24 weeks 3. MMSE significant difference between groups after 12 (*p*=0.047) and 24 weeks (*p*=0.031)
Turner et al. [53]	Phase II randomized, double-blinded, placebo-controlled single-site study	Subjects with mild to moderate AD (NIA-AA) Treatment group: male 3/female 14 Age 72.2 ±6.9 Placebo group: male 2/female 6 Age 69.2 ±6.06 Average MMSE N, 19.2 P, 19.8 CSF Aβ <1100pg/ml or positive amyloid PET	1:1 (*n*=37) 17 nilotinib 20 placebo Block randomization	Safety, tolerability Pharmacokinetics Effects on amyloid biomarkers on CSF Aβ42 and Aβ40, CNS amyloid burden [PET], CSF p-tau, total tau, and hippocampal volume (MRI) Clinical assessments (MMSE, ADAS-Cog, ADCS-ADL, NPI, CDR-SOB)	12 months	Nilotinib (150mg/d followed by 300mg/d)	SAEs 0% in the nilotinib group Mood swings (70.6%) mainly with 300mg/d dosage SAEs 25% in the placebo group	3 discontinued in placebo due to SAEs 3 voluntary discontinuation in nilotinib	1. Well-tolerated 2. Reduction in CNS amyloid burden and levels of CSF Aβ1-42, Aβ1-40, and p-tau with both dosages 3. Attenuation of hippocampal volume loss (−27%) 3. No significant efficacy in cognitive tests
Ghosal et al. [54]	Phase I Randomized Double-blinded Placebo-controlled proof-of-mechanism study	Healthy subjects (median age 30–32 y) all carrying ApoE ε3/ε3 Treatment group: female 6/male 0 Age 30.2±6.6 Placebo group: male 3/female 3 Age 32±9.6	1:1 (*n*=12) 6 bexarotene 6 placebo Atmospheric method for randomization	CNS penetration Increment of ApoE Alteration of Aβ Clearance	5 days	Bexarotene (450mg/d)	No SAEs were reported 3: increase triglyceride levels (>200 mg/ml) 1: increase cholesterol levels (>200mg/dl) 2: abnormal thyroid levels	No dropouts	1. Poor CNS penetration Bexarotene plasma to CSF ratio 85:1 2. No effect on clearance of Aβ
Decourt et al. [48]	Phase II, randomized double-blinded, placebo-controlled, single-site study	Male 16 (64%) Treatment group: male n.a./female n.a. Age 73.6 ±8.22 Placebo group: male n.a./female n.a. Age 73.6 ±4.84 Probable AD for at least 1 year (NINCD-ADRDA) Average MMSE T, 21.8 P, 22.0	2:1 (*n*=25) 17 thalidomide 8 placebo	Safety, tolerability ADAS-Cog ADCS-ADL, CDR-SOB, MMSE	24 weeks	Thalidomide (escalating dose regimens from 50 to 400 mg/d)	15/17 (88%) had AEs All AEs were reported for both arms	10/17 (67%) in the thalidomide arm terminated early 2/4 (50%) in the placebo group terminated early	1. Not well-tolerated, poor safety 2. Results on clinical outcomes were negative

#### Design and study population

Four studies [48, 51–53] enrolled patients with a diagnosis of mild to moderate AD while one study [54] recruited healthy volunteers; four studies enrolled patients older than 50 years [48, 51–53], while one study [54] recruited young volunteers [age range 21–50]. In two studies [52, 53], a positive amyloid PET was required as an additional criterion before randomization.

All five studies adopted a randomized, double-blind design. Only one study was a multicenter trial [51]. Allocation ratio, treatment duration, drug, and placebo doses were always described. Four trials [48, 52–54] adopted a two-arm design while the remaining one [51] relied on a multi-arm design. All studies reported that the appearance and way of administration of drug and matching placebo were identical. In some cases, packaging and labeling were generated and held by a third-party service to ensure a blinding procedure.

**Fig. 3:**
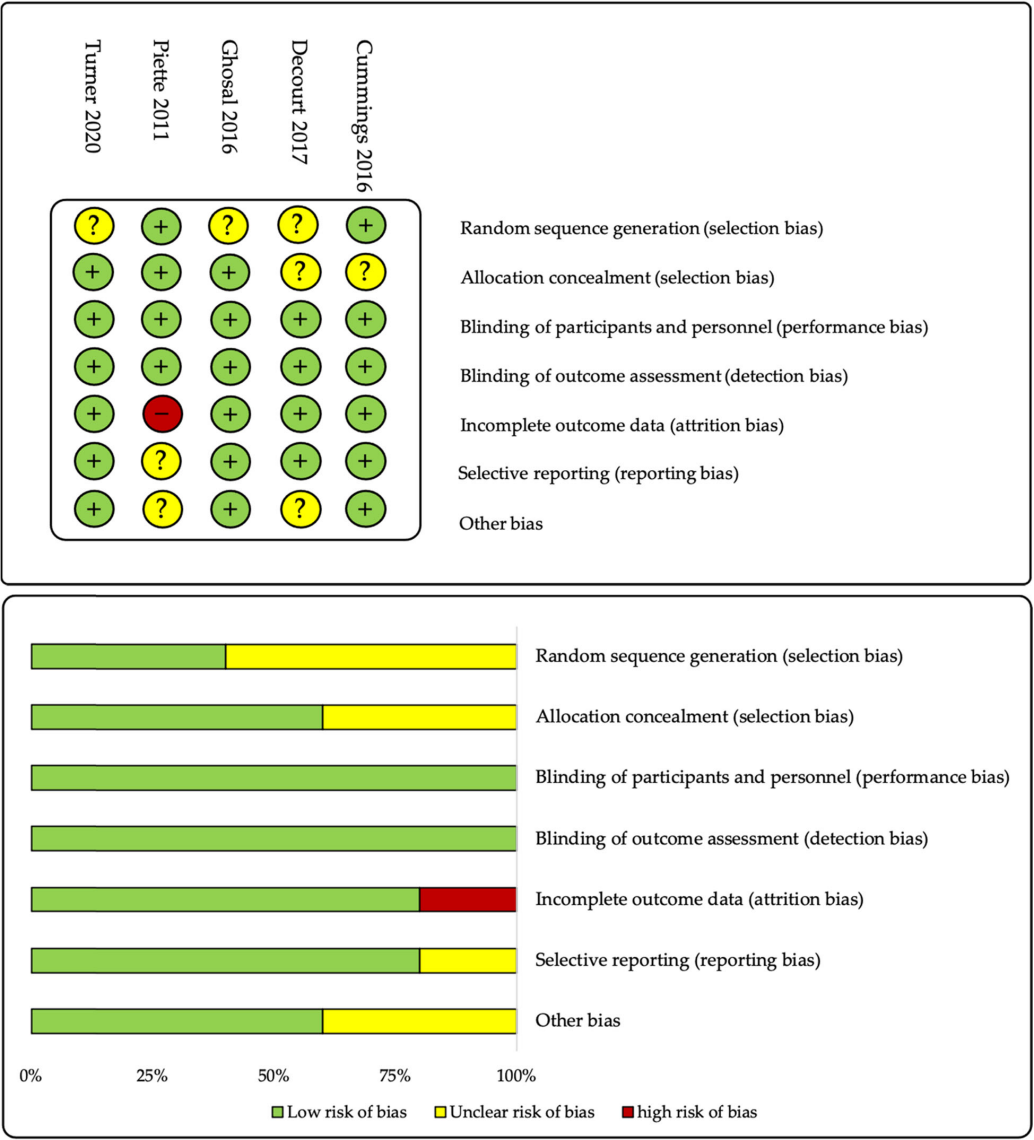
Risk of bias tool for methodological evaluation of published clinical studies

We used the RoB tool for quality analysis of randomized studies (Fig. 3). Our analysis of random sequence generation (selection bias) assessed that three studies had an unclear risk of bias [48, 53, 54] while, for two studies, a low risk was estimated [51, 52]. The enrollment and allocation process were reported in all studies. However, in two studies, the flow diagram of the randomization process was not available [48, 54]. Baseline characteristics and clinical features of participants were reported for both treatment and placebo groups in all studies. Only for one study [51], *p*-values were presented in tables to highlight between-group differences at the baseline. Only in two studies [52, 53], ethnicity was reported among baseline characteristics with white/Caucasian participants accounting for the overwhelming majority of participants (90–95%).

#### Apoliprotein E (ApoE) genotype

Information on ApoE genotype was reported only for three studies in summary tables [52–54]. In the nilotinib study, all ApoE genotypes identified in both treatment and placebo groups were reported. In the phase II bexarotene study, the frequency of ApoE-ε4 carriers (homozygotes and heterozygotes) and noncarriers was provided. In the phase I bexarotene study, based on theoretical concerns that the ApoE-ε4 allele may confer toxic gain of function and side effects, it was considered as appropriate to enroll only ApoE ε3/ε3 carriers. For two studies [48, 51], genotype profiles were not characterized.

#### Concomitant treatments and investigational drug dosages

Participants with AD were allowed to continue their treatments with cholinesterase inhibitors and/or memantine if on a stable dose. Investigational drugs were thus administered as adjunct therapies to the standard of care. Regarding drug dose, fixed dosages were assessed in bexarotene studies [52, 54]. Conversely, in the nilotinib trial [53], patients received escalating dose regimens unless safety and tolerability concerns appeared. In the masitinib study [51], blinded dose adjustments were allowed in the case of minimal toxicity or lack of response. In the thalidomide study [48], patients received escalating dose regimens previously adopted in oncological studies. Additional information on therapeutic regimens and posology is provided in Table 3.

#### Safety profiles

Safety analysis, drug tolerability, and AEs were reported for each study. A low risk of reporting bias was observed in four studies [48, 52–54]. In the masitinib study [51], only AEs with an incidence greater or equal to 5% were reported. A comparison between the masitinib’s safety profile observed in patients with AD and that emerged in other masitinib phase II non-oncology studies showed similar findings. A high discontinuation rate occurred in the treatment arm of the masitinib trial as compared to placebo (65% vs 25%). However, a similar frequency of severe AEs occurred was documented in the masitinib and placebo arms (15% vs 13% of patients, respectively). Moreover, only seven out of 26 subjects (27%) discontinued due to AEs while 10 subjects interrupted the treatment for reasons unrelated to the exposure. In the bexarotene studies [52, 54], increased triglyceride and cholesterol levels were observed both in healthy subjects and patients with AD. In the nilotinib study [53], the drug revealed an acceptable safety profile.

Poor safety was reported in the thalidomide study [48]. Based on our judgments, attrition bias was low in four studies [48, 52–54], since equal loss of participants occurred both in treatment and control arms.

#### Results for reported outcomes

Safety and tolerability were assessed as primary outcomes in three studies [48, 53, 54]. Biological outcomes associated with the reduction of CNS amyloid markers were evaluated in three studies [52–54]. In the proof-of-mechanism study [54], only low nanomolar levels of bexarotene were found in CSF and poor CNS penetration in the brain of healthy subjects was documented. However, the authors cautioned that the BBB of healthy human subjects would show lower permeability. The study on thalidomide [48] showed that poor safety and high toxicity hampered the use of a potentially therapeutic dose. Conversely, bexarotene, masitinib, and nilotinib showed more favorable safety profiles.

All four studies on patients with AD assessed cognitive and/or functional and/or neuropsychiatric changes through the administration of clinical tools (ADAS-Cog, ADCS-ADL, MMSE, MoCA, CDR-SB, CIBIC-Plus, and NPI). No study used a comprehensive neuropsychological test battery to measure cognitive modifications. Three studies [48, 52, 53] did not report any significant cognitive improvement, while the masitinib study [51] showed significant efficacy results measured with a decrease greater or equal to four points of the ADAS-Cog score at 12 and 24 weeks (6% of participants in the masitinib group experienced a cognitive decline as compared with 50% of those receiving placebo, *p*=0.040 and *p*=0.046, respectively).

Nilotinib achieved relevant CSF concentrations. Furthermore, it significantly reduced amyloid burden in the frontal lobe, measured by florbetaben PET at 12 months, and attenuated hippocampal volume loss. No significant result was observed for the explorative clinical outcomes.

## Discussion

To the best of our knowledge, the present study is the first attempt to systematically collect and discuss available data on the clinical use of approved anticancer agents in AD. Based on the present analysis, the possibility of modifying the AD pathophysiology and clinical course through the use of anticancer agents is increasingly investigated. The results of several randomized controlled trials have already been published and shared with the scientific community [48, 51–54], while further studies are currently underway and are expected to be completed in the next few years, thus generating additional evidence in the field.

Three out of five published randomized controlled trials, two bexarotene studies [52, 54] and a thalidomide study, [48] did not show any promising results, mainly for reasons related to toxicity and poor CNS penetration. Explorative clinical outcomes in the nilotinib [53] study showed promising results that should be confirmed in larger and longer studies. Masitinib was found to slow down the rate of cognitive decline in AD [51]. It is noteworthy that a larger phase IIb/III study on masitinib has recently been completed on more than seven hundred patients and, according to the statement of AB Science (the industry that developed the drug) [55], the drug met the primary endpoint by significantly improving both cognition and functional abilities. Although masitinib is currently approved for veterinary use, it is also currently under evaluation in humans for the treatment of diverse conditions including malignant melanoma, mastocytosis, multiple myeloma, gastrointestinal and pancreatic cancers, and multiple sclerosis [30].

Drug repurposing may consent to optimize the efforts to develop new treatments for AD by exploring the AD-related effects of agents already approved for other clinical indications [16]. This approach is promising since many approved pharmacological agents have shown AD-relevant effects in animal models. Moreover, it may significantly reduce the times and costs of drug development given that the repurposed drugs have already been tested in terms of safety/tolerability, thus rendering the conduction of further preclinical studies unnecessary [16]. In 2020, 53 clinical trials involving 58 FDA-approved agents acting on multiple therapeutic targets (e.g., neuroinflammation, neuroprotection, neurotransmitter modification) were registered in the ClinicalTrials.gov database, accounting for 39% of the overall AD pipeline [16]. In parallel, since 2019, the number of phase III studies targeting Aβ dropped by 20% [56].

In the last decades, in vitro and animal studies have provided promising evidence supporting the repurposing of anticancer agents for AD [21, 26, 57, 58]. In particular, agents acting as TKIs are attracting special attention. Emerging evidence justifies TKI utilization in AD [26–31, 33–41]. The inhibition of several kinases has been associated with lower Aβ deposition and tau phosphorylation [26, 57] and hampered amyloidogenic APP processing in AD neurons [58]. RXR agonists have also provided promising preclinical results [42–45]. Particularly, bexarotene was found to enhance the clearance of soluble Aβ within hours in an ApoE-dependent manner, to inhibit Aβ42 aggregation and reduce neuroinflammation, and to revert cognitive deficits [42–44] (Table 2). These promising preclinical results were not confirmed in humans mainly due to poor CNS penetration and deficient cerebrospinal fluid concentrations. Moreover, frequent serious AEs (i.e., elevated triglycerides) were observed [54]. Other anticancer drugs such as thalidomide, lenalidomide, and pexidartinib have been shown to exert neuroprotective effects and attenuate neuroinflammation in experimental models [37–40, 46, 59]. Masitinib, as well, showed promising anti-neuroinflammatory effects through the modulation of microglia and amyloidosis, or with a synaptoprotective action in relation with mast cell inhibition [30–34]. Overall, targeting several actors implicated in neuroinflammation, together with the reduction of brain amyloid burden, currently represents the primary therapeutic rationale for the repurposing of anticancer drugs in AD [49, 50, 60–62].

Promisingly, most of the completed and ongoing clinical studies testing anticancer agents in the continuum of AD are adopting a randomized, placebo-controlled design. Moreover, a sizeable proportion of these protocols is already assessing meaningful clinical outcomes (e.g., cognitive and functional improvement) besides exploring the safety/tolerability profiles of the investigational interventions and their effects on specific biomarkers. These methodological features enhance the clinical relevance of the findings that will emerge from these trials. At the same time, much remains to be done in this field. Moreover, several methodological shortcomings still limit the overall quality of the available evidence. Indeed, most studies are recruiting very small populations of patients, with heterogeneous clinical manifestations (e.g., at different dementia stages); are conducted in single clinical sites; and are at the earlier phases of drug development.

Several limitations of the present study are worth to be acknowledged and discussed. First, besides ClinicalTrials.gov and EudraCT, there are other registries for research protocols (in particular, for those conducted outside the USA and EU). Therefore, our study should not be regarded as an exhaustive overview on the topic. Moreover, such databases only collect a limited amount of data on the methodology of the ongoing studies. In addition, eventual protocol amendments and updates may not be timely reported. A further limitation of the present study is the lack of a quantitative analysis of the reviewed evidence. However, identified studies did not focus on the same research question and adopted different methodological designs (e.g., different disease severity, interventions, comparators, and outcomes), thus hampering the conduction of a metanalysis and quantitative comparisons. On the contrary, the main strength of this study is the choice of merging available evidence coming from both ongoing research protocols and completed clinical trials. This approach has allowed us to provide a comprehensive perspective on the repurposing of anticancer agents for AD. However, to have an exhaustive overview of the efficacy and safety of anticancer drugs currently underway for AD, we encourage the scientific community to disclose trial data, even when results do not seem promising, thereby preventing publication bias.

## Conclusions

In conclusion, based on the present overview, the repurposing of anticancer agents for the treatment of AD is triggering growing interest. The promising results emerging from preclinical studies and identified research protocols should be confirmed and extended by larger, adequately designed, and high-quality clinical trials.

## Data Availability

The datasets used and/or analyzed during the current study are available from the corresponding author on reasonable request.

## Abbreviations

Αβ: Amyloid-beta
AD: Alzheimer’s disease
ADAS-Cog: Alzheimer’s Disease Assessment Scale–Cognitive Subscale
ADCS-ADL: Alzheimer’s Disease Cooperative Study-Activities of Daily Living
AE: Adverse event
ApoE: Apoliprotein E
BBB: Blood-brain barrier
CIBIC-Plus: Clinician’s Interview-Based Impression of Change Plus Caregiver Input
CDR-SB: Clinical dementia rating–sum of boxes
CNS: Central nervous system
CSF: Cerebrospinal fluid
MCI: Mild cognitive impairment
MMSE: Mini Mental State Examination
MoCA: Montreal Cognitive Assessment
NPI: The Neuropsychiatric Inventory
SAE: Serious adverse event
TKI: Tyrosine kinase inhibitor
